# Plasmatic markers in hemorrhagic stroke

**Published:** 2011-05-25

**Authors:** IC Marginean, DM Stanca, V Vacaras, O Soritau, M Margiean, DF Muresanu

**Affiliations:** * Department of Neurology, University of Medicine and Pharmacy, Cluj–NapocaRomania; ** Department of Cancer Immunology of ‘Prof Dr Ion Chiricuta’ Comprehensive Cancer CenterRomania; *** Department of Histology, University of Medicine and Pharmacy, Cluj–NapocaRomania

**Keywords:** biomarker, stroke, hemorrhagic stroke, ischemic stroke, diagnosis

## Abstract

Stroke is the third most common cause of death in the United States and it is the leading cause of disability. Early diagnosis and immediate therapeutic interventions are important factors to reduce the extent of brain tissue damage and the risk of stroke–related death. A rapid blood test that can confirm the clinical or imaging diagnosis or that can add to the stratification of the risk would be very useful. Such a test has to be validated in large studies and has to be based on a simple and low–cost technology. Many biological markers were tested for their ability to serve as ‘would–be’ stroke biological markers; some of them appear to have a place in the diagnostic work–up of stroke patients. These molecules include Glial Fibrillary Acidic Protein (GFAP), the N–methyl–D–aspartate receptor (NMDA), APO C–III, APO C–I, PARK7, nucleoside diphosphate kinase A (NDKA), S100B, B–type neurotrophic growth factor, von Willebrand factor, matrix metalloproteinase–9, and monocyte chemotactic protein–1. There are obvious limitations to this study, among them the fact that disability does not necessarily correlate with the amount of cerebral tissue lost (the site of stroke may be more important) and the role of the blood–brain barrier in delaying the release of the neuronal proteins in the blood stream. Further studies are awaited to confirm the role of these molecules in the management of acute stroke patients.

## Introduction

Stroke is the third most common cause of death in the United States and the leading cause of disability (physical and mental). The survivors of such an event are confronted with physical and mental handicaps (5 years from the acute event up to 25% of stroke patients develop vascular dementia), with a decisive impact on life expectancy, quality of life and the cost supported by the society. The health–care expenditures resulting from a stroke are estimated to be 140,000 dollars per patient [1]. Chronic nursing costs can be reduced by limiting the number of sequelae by a more precocious diagnosis during the acute period, thus allowing initiation of a specific therapy even before the patient reaches the stroke unit, and by adaptation of the therapy to the risk of each patient.

At the moment, the diagnosis of stroke is based on a stroke clinician's examination of the patient, supplemented by the results of brain imaging. However, in people who are suspected of having a stroke, the clinical assessment within the first hours is not always straightforward. Either patients with acute stroke cannot be assessed by a stroke specialist, or the interpretation of the brain images can be difficult, as computerized tomography (CT) images are often normal after the onset of ischemia and may remain normal in patients with mild ischemic strokes. MRI is still not 100% sensitive or specific and may not be feasible in acutely ill patients because they are restless, they have a contraindication to MRI, or MRI is not immediately available. In the case of a hemorrhagic stroke, the early onset of a specific therapy may limit the extent of brain damage in the first hours. Thus, sending patients directly to specialized units (neurosurgery, stroke unit) may improve their prognoses [2].

A rapid blood test that can confirm the clinical or imaging diagnosis or can add to the stratification of the risk would be very useful. Such a test must be validated by large studies and must be based on a simple and low–cost technology. There are obvious limitations for such a study, among them the fact that disability does not necessarily correlate with the amount of cerebral tissue lost (the site of stroke may be more important) and the role of the blood–brain barrier in delaying the release of the neuronal proteins in the blood stream.

The ability of many biological markers to serve as ‘would–be’ stroke biological markers was studied; some of these molecules appear to have a place in the diagnostic work–up of the stroke patient. These molecules include Glial Fibrillary Acidic Protein (GFAP), the N–methyl–D–aspartate receptor (NMDA), APO C–III, APO C–I, PARK7, nucleoside diphosphate kinase A (NDKA), and S100B [3]. All of these molecules appear to have significance for ischemic stroke; GFAP, S100B and APO C–III may also be used as potential plasmatic markers to distinguish between ischemic and hemorrhagic stroke. Reynolds developed a panel of five proteins (S100B, B–type neurotrophic growth factor, von Willebrand factor, matrix metalloproteinase–9, and monocyte chemotactic protein–1) that appears to be capable of diagnosing a stroke with sensitivity and specificity above 90% [4].

### Glial Fibrillary Acidic Protein (GFAP)

GFAP is a brain–specific intermediate filament protein found in astrocytes. It was recently identified as a biomarker indicative of intracerebral hemorrhage in the acute phase of stroke. Foerch prospectively included 93 patients with ischemic stroke (IS) and 42 patients with intracerebral hemorrhage (ICH) within 6 hours of symptom onset. GFAP was detectable in the serum in 81% of ICH patients but only in 5% of IS patients. The mean GFAP serum concentration was significantly higher in patients with ICH. A cutoff point of 2.9 ng/L was found to provide a sensitivity of 79% and a specificity of 98% for the differentiation of ICH from IS. The more sudden disruption of astroglial cells and the blood–brain barrier (BBB) in ICH may be responsible for the rapid occurrence of GFAP in the serum, in comparison to a more delayed release of astroglial proteins in IS [5].

### S100B

S100B is a Ca^2+^–binding protein found in glial cells in the central nervous system. The concentration of this protein increases in the plasma after brain damage due to IS; high blood S100B concentrations are associated with infarct size, outcome and neurovascular status on admission [6]. It also can be used as a marker of success after thrombolysis with a tissue plasminogen activator.

More recent studies have found increased S100B plasma levels after acute spontaneous ICH in association with worse early and late clinical evolution. Moreover, S100B is considered a relevant diagnostic tool in the perinatal context due to its ability to predict intraventricular hemorrhage in preterm and asphyxiated full–term infants when other clinical or radiological assessments are still silent [7, 8].

### APO C–III

ApoC–III is present in normal plasma at 0.1 g/L and is mainly found in VLDL but is also found in HDL and LDL particles. It is mainly expressed by the liver. ApoC–III is known to inhibit lipoprotein lipase present on the surface of vascular endothelial cells. A high ApoC–III concentration in lipoproteins is a prominent component of atherogenic dyslipidemia and explains the risk of coronary heart disease associated with high triglyceride levels. APO C–III is overexpressed in acute IS patients relative to acute ICH patients [9].

## Conclusion

Early diagnosis and immediate therapeutic interventions are important factors to reduce the extent of brain tissue damage and the risk of stroke–related death. Currently, the diagnosis of stroke relies on neurological assessment of the patient and neuro–imaging techniques including computed tomography and/or magnetic resonance imaging. An early diagnostic marker of stroke, ideally capable of discriminating ischemic stroke from hemorrhagic stroke, would considerably improve patient management. There are some biological markers that have been studied as predictors of the type of stroke, including GFAP, NMDA, S100B, and Apo C–III. Further studies are awaited to confirm the role of these markers in the management of patients with acute stroke.

This work was supported by CNCSIS–UEFISCSU, project number PNII–IDEI 2621/2008.
